# A retrospective study: efficacy of an originator versus a generic formulation of simvastatin in patients who suffer from hyperlipidaemia

**DOI:** 10.5830/CVJA-2022-053

**Published:** 2022-12-08

**Authors:** JR Snyman, KR Snyman

**Affiliations:** Clinical pharmacologist, Pretoria, South Africa; Pharmaceutics, Cape Town, South Africa

**Keywords:** hyperlipidaemia, simvastatin, cholesterol, lipid, hypercholesterolaemia

## Abstract

**Background:**

South Africa is home to a multi-ethnic society with a large range of cultures and lifestyles. Cardiovascular disease is a major cause of morbidity and mortality. South Africa is known to have one of the highest incidence rates of hypercholesterolaemia in the world, especially among the Caucasian population.

**Aim:**

The aim of this retrospective chart review was to establish whether a multisource simvastatin (Simvotin®, Ranbaxy, a Sun Pharma company) maintained the cholesterol-lowering effect after switching from the innovator brand Zocor® (MSD South Africa) in the public-sector hospitals. Since prescribers often doubt the registration requirements of multisource products based on bioequivalence alone, this research was done to confirm similar clinical outcomes in a real-world setting.

**Methods:**

More than 200 charts were identified from patients treated for hyperlipidaemia. Patients were treated for at least six months prior to and again six months after the switching of brands in order to meet criteria to be eligible for inclusion. The lipid values at initiation of therapy as well as before switching (visit 1 and 2) had to be available and again six months after treatment on the multisource product (visit 3).

**Results:**

No significant change was observed in the lipid control after switching, confirming similarity.

**Conclusion:**

This real-world evidence should allay any fears of generic inferiority of this important medicine in the treatment and prevention of high cardiovascular risk in patients requiring lipid-lowering therapy.

This study investigated the originator and generic formulations of simvastatin to determine whether clinical equivalence exists in a real-world setting. This study was undertaken in order to investigate whether there was indeed clinical or therapeutic equivalence between the originator and generic formulations of simvastatin, researching treated patient data retrospectively, before and after switching between two branded versions of simvastatin, one the originator and the other a multisource equivalent.

In practice, the most important and common form of dyslipidaemia is hypercholesterolaemia.^1^ Research from animal and laboratory investigations, epidemiology and genetic forms of hypercholesterolaemia indicate that an elevated low-density lipoprotein cholesterol (LDL-C) level is a major cause of coronary heart disease (CHD).^2^

The South African guidelines advocate that the use of drug therapy must balance the cost against the clinical efficacy and risk of CHD.^1^,^3^ The recommendations, when initiating drug therapy, are indicated as follows: lipid-lowering drugs should be recommended to patients with a 10-year risk of an overt CHD event of > 20%, projected to the age of 60 years.^4^ Although clinical judgement plays an important role in initiation of treatment, patients with familial hypercholesterolaemia or the presence of established CHD (including a previous acute myocardial infarction) should be candidates for lipid-lowering drugs.

The response to medicinal interventions varies from person to person and the general consensus is that an ‘anti-coronary’ diet, weight loss as well as a regular exercise routine are able to enhance the effects of lipid-lowering agents significantly.^1^,^3^ One of the pharmacological interventions used to treat increased cholesterol levels is a class of drugs called the statins. Statins are by far the most prescribed treatment choice for LDL-C reduction, which demonstrably reduce cardiovascular mortality rate.^5^

Statins act by inhibiting hydroxy-methylglutaryl co-enzyme A reductase, a key enzyme in cholesterol synthesis, leading up to an increased LDL-C clearance. The statins reduce LDL-C levels by up to 60% and produce small increases in highdensity lipoprotein cholesterol (HDL-C) and triglycerides (TG).

Statins also appear to decrease intra-arterial and/or systemic inflammation by stimulating production of endothelial nitric oxide.^5^ This class of drug may also decrease LDL-C deposition in endothelial macrophages as well as decrease cholesterol in inflammatory cell membranes. This anti-inflammatory effect is anti-atherogenic, even in the absence of elevated lipid levels.^5^

Evidence from large-scale, prospective, double-blind, randomised clinical trials clearly indicates a rate reduction in total and cardiovascular morbidity and mortality with statins, initially for pravastatin,^6^,^7^ and shortly followed by evidence for simvastatin.^8^,^9^ An average reduction in LDL-C of about 1 mmol/l, maintained for approximately five years, produced a reduction in non-fatal myocardial infarction and coronary death in about one-quarter of the study populations.^9^

Although the concept of aggressive treatment of raised LDL-C level is now well entrenched, the cost of long-term treatment is often seen as prohibitive by third-party funders, especially in lower-risk individuals, therefore the increase in entry threshold of treatment. The introduction of multisource generic products has however, eroded this objection but introduced yet another from the prescriber and that is distrust in the equivalence of products.

In order to investigate whether the distrust is founded on evidence, it was decided to retrospectively investigate the impact of the introduction of a multisource product into public-sector (government funded) lipid clinics by evaluating LDL-C values over time, before and after introduction. The primary aim of this real-world study was, therefore, to assess the efficacy of a generic formulation of simvastatin (SimvotinR, Ranbaxy, a Sun Pharma company) compared to a non-generic (originator) formulation of simvastatin (ZocorR, MSD South Africa) in patients with hyperlipidaemia, by assessing the maintenance of lipid control after the switch of medication from ZocorR to SimvotinR.

## Methods

The analysis was performed as a multi-centre, retrospective study at three sites in South Africa (one each in Gauteng, Free State and KwaZulu Natal provinces). A retrospective, short review of patient files was done with no administration of study treatment.

**Fig. 1:**
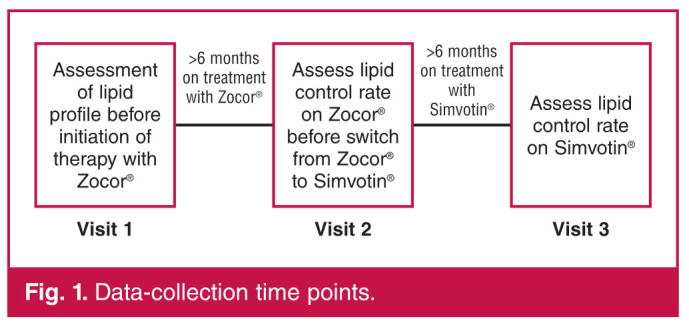
Data-collection time points.

Ethics committee approval was obtained for retrospective chart review at each centre prior to initiation of the study and data collection. Patients received their normal care and medication at these public lipid or cardiology clinics. The study focused on a time period from 2005 to 2007, during which the simvastatin dispensed to patients at these clinics changed from the original drug (ZocorR) to the generic drug (SimvotinR). The time points for data collection assessed is illustrated in Fig. 1.

The patients included in this study were male and female between the ages of 18 and 80 years who had been on treatment with the originator drug (ZocorR) for at least six months on the same dosage, before being switched to the generic drug (SimvotinR), and where the dose had been unchanged for the period until the second evaluation, which was at least six months after the switch. Patients were excluded from the study if they had been taking any other lipid-lowering agents in addition to the simvastatin. Demographics of the patients are presented in Table 1.

**Table 1:** Patient demographics

*Parameters*	*Number (%) of patients*
Male	87 (42.4)
Female	118 (57.6)
Total	205 (100)
Non-smoker	156 (76.1)
Smoker	39 (19.0)
Ex-smoker	10 (4.9)
Total	205 (100)

This study was a retrospective chart review of patients’ files and therefore required no study-related administration of treatment. The lipid-lowering agents in treatment that were evaluated were simvastatin oral tablets, namely ZocorR and SimvotinR tablets that were used in a once-daily dosing regimen.

No formal sample size estimation was done. A sample of 205 patients from three sites was enrolled into the study. These individuals formed the total number of patients who fulfilled the stated criteria and had pathology reports on lipid profiles on file. Patient were excluded if the lipid profiles were not available, or if they were taking other lipid-lowering agents, or were non-compliant (missed more than one script fill in a six-month period). All patients had to switch from originator to generic multisource product to be included in the study.

With a sample size of 205 patients switched from ZocorR to SimvotinR, a two-sided 95% confidence interval for the true proportion (percentage) of patients controlled (the control rate) after the switch, using the large sample normal approximation of the binomial distribution, will extend to approximately 0.03 (3%) from the observed proportion (percentage) to be calculated from the sample, if the expected proportion (percentage) of controlled patients (after the switch) is between 0.85 and 0.90 (85 to 90%).

The primary outcome of the study was to assess the efficacy of the generic formulation SimvotinR tablets in controlling the total cholesterol level in patients in relation to baseline (visit 1) after being switched from ZocorR tablets. All lipid levels available were recorded and analysed as secondary outcomes. They are displayed in Table 2.

**Table 2:** Cholesterol values (mmol/l): patients were on a constant strength of simvastatin between the time points

*Visit*	*Number*	*Mean*	*SD*	*Mean change*	*p-value*
Total cholesterol					
Visit 1	117	6.28	4.35		
Visit 2	118	5.43	1.65		
Visit 3	118	5.53	1.65		
AV1->22	117	-0.84	4.16	-0.84	0.030*
AV2->33	118	0.10	1.00	0.10	0.283
HDL-C					
Visit 1	117	1.16	0.37		
Visit 2	118	1.18	0.41		
Visit 3	117	1.15	0.44		
AV1->22	117	0.02	0.28	0.02	0.515
AV2->33	117	-0.02	0.28	-0.02	0.426
LDL-C					
Visit 1	113	3.90	1.85		
Visit 2	115	3.55	1.74		
Visit 3	114	3.67	1.68		
AV1->22	111	-0.39	1.04	-0.39	< 0.001*
AV2->33	112	0.10	0.86	0.10	0.202
Triglycerides					
Visit 1	41	1.81	1.05		
Visit 2	118	1.66	1.17		
Visit 3	118	1.63	0.94		
AV1->22	41	-0.26	1.08	-0.26	0.127
AV2->33	118	-0.03	1.05	-0.03	0.753

* Statistically significant. LDL-C, low-density lipoprotein cholesterol; HDL-C, high-density lipoprotein cholesterol; ΔV1→V2, difference between visit 1 and visit 2.

## Statistical analysis

All statistical procedures were performed on SASR, release 9.1, run under MicrosoftR WindowsR for a personal computer, and p-values ≤ 0.05 were considered significant.

## Results

The charts of 205 patients who fulfilled the inclusion criteria were included for review in the study. Of the 205 patients enrolled, 118 patients had the same strength of simvastatin administered at all three data-collection points (visit 1, 2 and 3), which means they had been prescribed the same strength of statin even after the switch from ZocorR to SimvotinR tablets (Table 2). These 118 patients would then not have a dosedifferent effect on cholesterol levels, which will then be used to demonstrate similarity. Dose continuity was a requirement for therapeutic equivalence.

The data were collected at the three time points on case report forms with specified fields captured to determine the outcome of the study. Among the fields assessed were total cholesterol, HDL-C, LDL-C (mmol/l, fasting) and TG (mmol/l, fasting).

The total cholesterol, HDL-C, LDL-C and TG levels are reflected in Table 2. The mean changes in these levels from visit 1 to visit 2 (the reference period) and from visit 2 to visit 3 (the maintenance period) are also shown. The results show that from visit 2 to visit 3 there was no significant change in total cholesterol levels and that SimvotinR tablets maintained lipid control in these patients, after the switch was made from ZocorR tablets. The switch between brands occurred between visit 2 and visit 3 and we allowed six months between these visits on the same drug dose to assess therapeutic similarity.

## Discussion

In this study, the generic simvastatin (SimvotinR) has been shown to be clinically equivalent in maintaining the lowered lipid levels at the same level as the originator simvastatin, ZocorR, in this real-world study.

Adequate risk-factor control in patients at high risk for cardiovascular disease is essential to improve long-term prognosis. The significant benefit of good risk-factor control has been well established in patients with hypercholesterolaemia. Statins or HMG-CoA (3-hydroxy-3-methylglutaryl co-enzyme A) reductase inhibitors still remain the backbone of the treatment of patients with hypercholesterolaemia and have been well proven to reduce mortality and morbidity rates.^10^

As the statin drugs, such as simvastatin, have come off-patent, multiple multisource products or so-called generic drugs have been introduced into the current market. Since clinical or therapeutic comparative studies have never been a prerequisite for registration of small-molecule oral generic drugs where the active product ingredient is identical and where bioequivalence alone is considered sufficient proof of equivalence by the relevant regulatory authorities, many clinicians have had doubts as to the clinical equivalence of these multisource generic medicines. In this study the multisource simvastatin (SimvotinR) has been shown to be therapeutically equivalent in maintaining the lowered lipid levels at the same level as the originator simvastatin (ZocorR).

A multisource product is described as having the same active product ingredient at the same dose strength, the same route of administration and same manufacturing quality and rigour, with a presumed similar therapeutic outcome. This then constitutes a bioequivalent product, according to the USA Food and Drug Administration (FDA).11 This real-world study aimed to demonstrate that a multisource product retained the therapeutic effect achieved by the originator.

Statins are reversible inhibitors of the HMG-CoA enzyme, which leads to improved cholesterol clearance due to upregulation of receptors on the hepatocytes (LDL-C receptors).^12^-^14^ The bioavailability of simvastatin is very low due to its extensive pre-systemic elimination (approximately 7%), which gives relevance to doing a therapeutic equivalence study ensuring real-world similarity, since a typical bioequivalence study may be impaired by low and variable bioavailability of simvastatin.^15^ With the appropriate evidence of clinical equivalence, this generic drug can be prescribed with confidence by the practitioner.

A generic drug is referred to as a drug that is a copy of a brand-name drug that has equivalent dosage, intended use, effectivity, side-effect profile, route of administration, risks, safety, as well as strength as the original drug and would, after dosing, reach the receptor site in equivalent concentrations and time exposure. Therefore, the pharmacological action is identical to that of the brand-name counterpart.^16^

Clinicians often express concern regarding therapeutic equivalence of multisource products. However, the fact that these products are more affordable and therapeutically equivalent may save the funder significantly and may enhance access.^17^ Pharmaceutical regulators around the world, such as the South African Health Products Regulatory Agency (SAHPRA) and the USA FDA requirements of a generic drug is that it is as safe and effective as the brand-name innovator.^16^,^18^

The efficacy and source of any medicine, in this case simvastatin, must be reliable to produce the desired clinical surrogate outcome, lowering of cholesterol, to be able to assume the same clinical risk-reduction outcome. Lipid lowering and reduced progression to significant coronary artery disease are the markers for successful treatment of hypercholesterolaemia and coronary artery disease.

Lifestyle changes may affect outcomes, which is often seen as a weakness of real-world studies. However, in this study no significant changes in mean weight were seen for the study subjects over the three data-collection time points, implying no major lifestyle impacts on the observed results. Other weaknesses of the study include the paucity of long-term outcomes, especially as these patients also suffered from other co-morbidities. However, the aim of the study was simply to assess therapeutic equivalence related to reduction and maintenance of reduction of lipid levels on different brands of simvastatin over a short period of time.

## Conclusions

The importance of established data illustrating bioequivalence of generic products when compared to the innovator product has become increasingly scrutinised by the medical community at large, who view bioequivalence data merely as a requirement to register a medicine in South Africa and not a marker for efficacy of the medicine. However, data illustrating the effectiveness in patient populations carry more weight in determining the acceptance of a product in the prescriber market. This retrospective, real-world analysis showed the effectiveness of a generic form of simvastatin (SimvotinR) in controlling and maintaining lipid levels in those patients who were switched from the innovator brand (ZocorR).

## References

[1] (2000). South African Medical Association and Lipid and Atherosclerosis Society of Southern Africa Working Group. Diagnosis, management and prevention of the common dyslipidaemias in South Africa – clinical guideline, 2000. Expert Rev S Afr Med J.

[2] National Cholesterol Education Program. Detection, Evaluation and Treatment of High Blood Cholesterol in Adults (Adult Treatment Panel III) Executive Summary. NIH publication no. 01-3670 May 2001: 1-28.

[3] Klug EQ, Raal FJ, Marais AD, Smuts CM, Schamroth C, Jankelow D, Blom DJ, Webb DA (2018). South African Dyslipidaemia Guideline Consensus Statement 2018 Update.. Expert Rev S Afr Med J.

[4] Pyorala K , De Backer G, Graham I, Poole-Wilson P, Wood D (1994). Prevention of coronary heart disease in clinical practice. Recommendations of the task force of the European Society of Cardiology, European Artherosclerosis Society and European Society of Hypertension.. Eur Heart J.

[5] Endocrine and metabolic disorders: dyslipidemia. www.merck.com.

[6] Shepherd J, Cobbe SM, Ford I, Isles CG, Lorimer AR, MacFarlane PW, McKillop JH, Packard CJ (2005). Prevention of coronary heart disease with pravastatin in men with hypercholesterolaemia.. New Eng J Med.

[7] Sacks FM, Pfeffer MA, Moye LA, Rouleau JL, Rutherford JD, Cole TG (1996). The effect of pravastatin on coronary events after myocardial infarction in patients with average cholesterol levels.. New Eng J Med.

[8] (1994). Scandinavian simvastatin survival group. Randomised trial of cholesterol lowering in 4444 patients with coronary heart disease: the Scandinavian Simvastatin Survival Study (4S).. Lancet.

[9] (2002). Heart Protection study collaborative group. Heart protection study of cholesterol lowering with simvastatin in 20536 high-risk individuals: a randomised placebo-controlled trial.. Lancet.

[10] Ward NC, Watts GF, Eckel RH (2019). Statin toxicity: mechanistic insights and clinical implications.. Circ Res.

[11] FDA. Generic Drug Facts. 2021.

[12] Dainis AM, Ashley  EA. (2018). Cardiovascular precision medicine in the genomics era.. J Am Coll Cardiol Basic Transl Sci.

[13] Sturm AC, Knowles JW, Gidding SS, Ahmad ZS, Ahmed CD, Ballantyne CM (2018). Convened by the Familial Hypercholesterolemia Foundation. Clinical genetic testing for familial hypercholesterolemia.. J Am Coll Cardiol Scien Expert Panel.

[14] Mytilinaiou M, Kyrou I, Khan M, Grammatopoulos DK, Randeva HS (2018). Familial hypercholesterolemia: new horizons for diagnosis and effective management.. Front Pharmacol.

[15] Mauro  VF (1993). Clinical pharmacokinetics and practical applications of simvastatin.. Clin Pharmacokinet.

[16] Stoppler M Generic drugs, are they as good as brand names? Available at: https://www.medicinenet.com/generic_drugs_are_they_as_good_as_ brand-names/views.htm. https://www.medicinenet.com/generic_drugs_are_they_as_good_as_brand-names/views.htm.

[17] Larmour I, Pignataro S, Barned KL, Mantas S, Korman MG (2011). A therapeutic equivalence program: evidence-based promotion of more efficient use of medicines.. Med J Aust.

[18] Kenned A (2017). Generics vs original medication.. https://fpm.co.za/2017/05/03/generic-vs-original-medication/#:~:text=Generic%20medicines%3A%20These%20medicines%20are,is%20also%20exactly%20the%20same.

